# Supplementary material for: Lamin regulates the dietary restriction response via the mTOR pathway in Caenorhabditis elegans
Source: J Cell Sci. 2021 Sep 6;134(17):jcs258428. doi: 10.1242/jcs.258428 (PMC8445603; doi:10.1242/jcs.258428)
Supplement: Supplementary information [file joces-134-258428-s1.pdf]

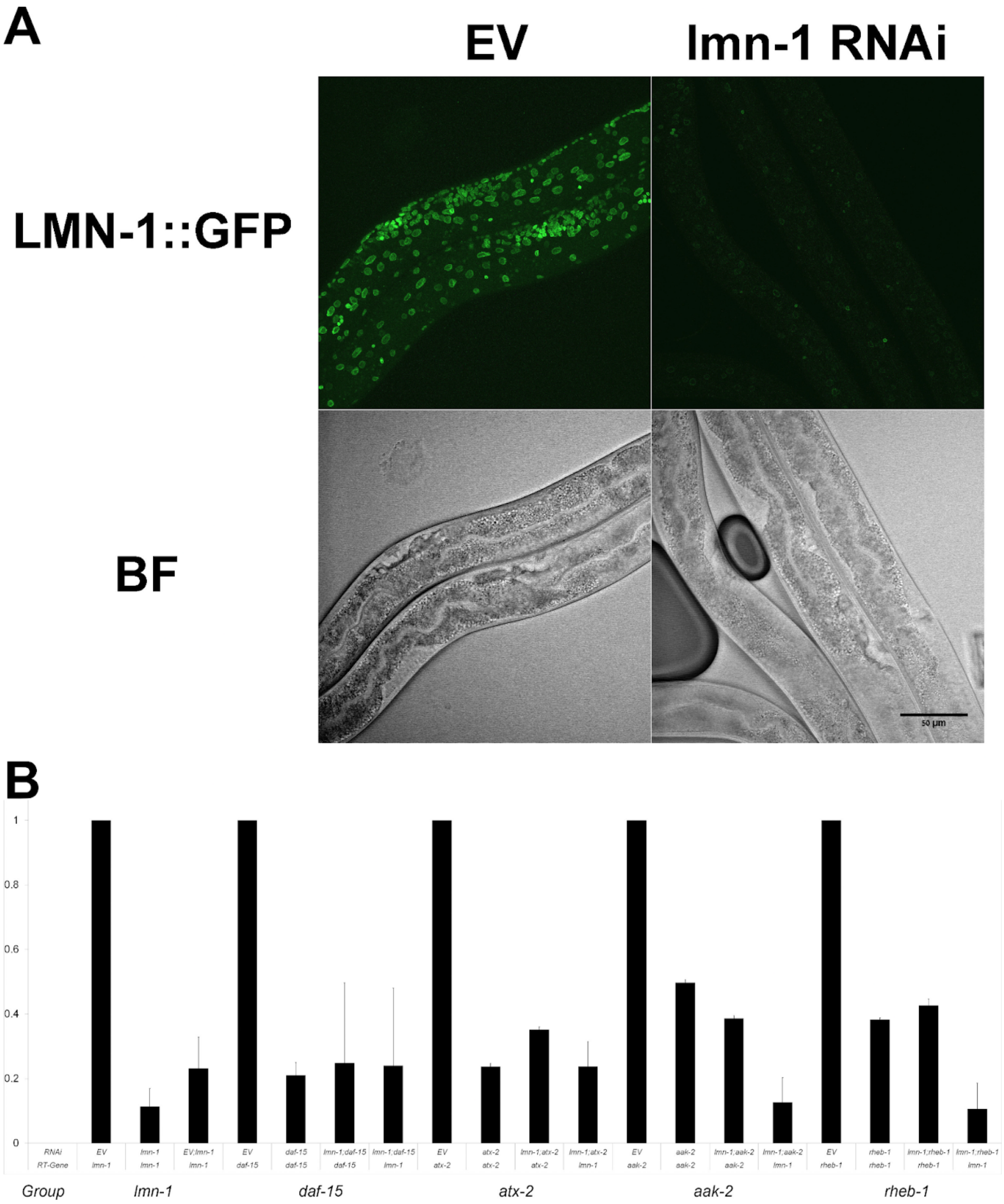

Fig. S1. (A) *lmn-1*(RNAi) successfully downregulated lamin, as evident from confocal microscopy of LMN-1::GFP animals. (B) Real time PCR quantification of gene levels following RNAi.

Journal of Cell Science • Supplementary information

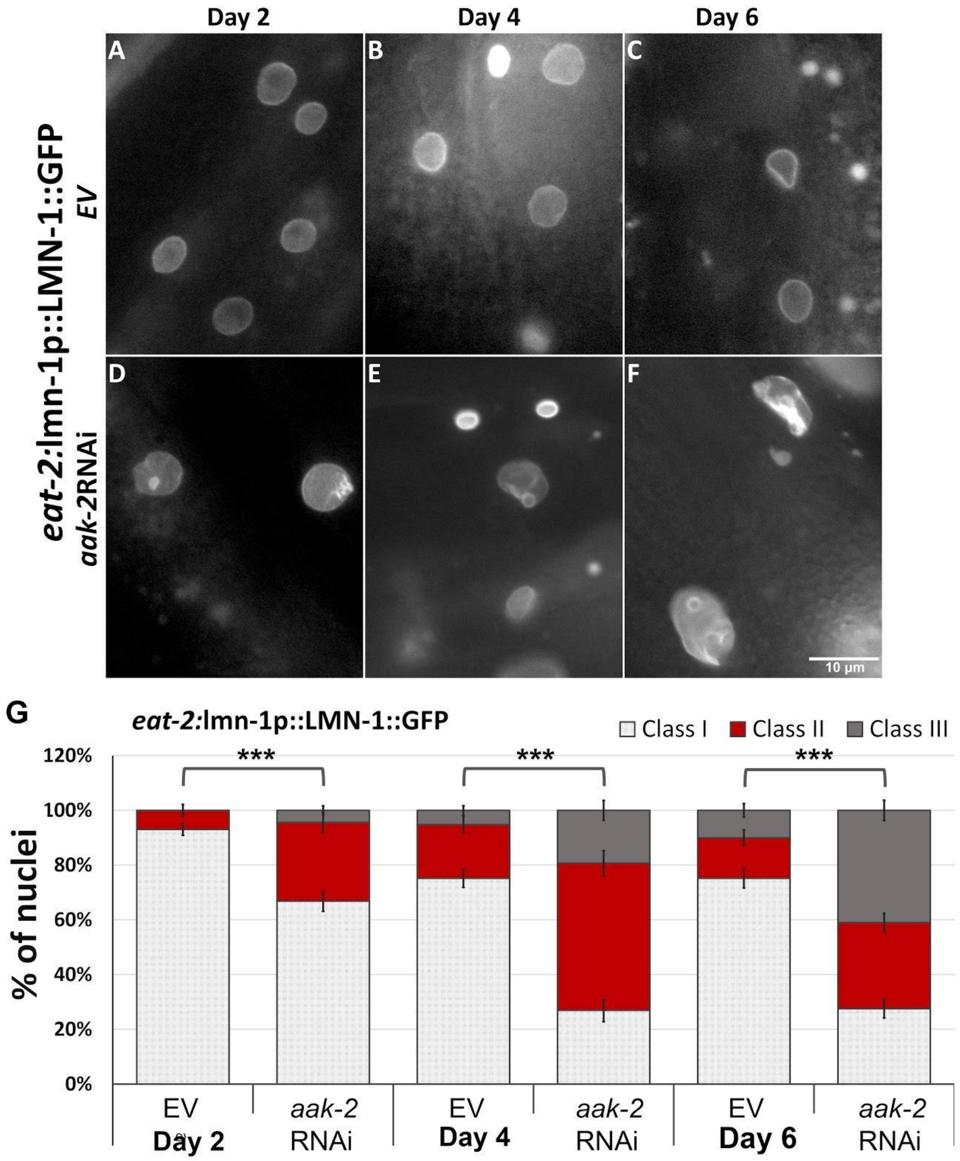

Fig. S2. AAK-2 is required to maintain nuclear structure (*A*-*F*) Microscope images of *eat-2* worms expressing LMN-1::GFP fed by either EV (*A*-*C*) or *aak-2* (RNAi) post-hatching (*D*-*F*) at day 2 (*A*,*D*), 4 (*B*,*E*) and 6 (*C*,*F*) of adulthood. (*G*) Relative distribution of the 3 different classes grading the nuclear morphology changes in eat-2 worms expressing LMN-1::GFP fed with *aak-2* (RNAi) or EV at days 2,4 and 6 of adulthood. n(D2)=303 nuclei, n(D4)=292 nuclei, n(D6)=327 nuclei. Error bars in all graphs represent mean ± SEM. P value was calculated using Fisher Exact Probability Test.

Journal of Cell Science • Supplementary information

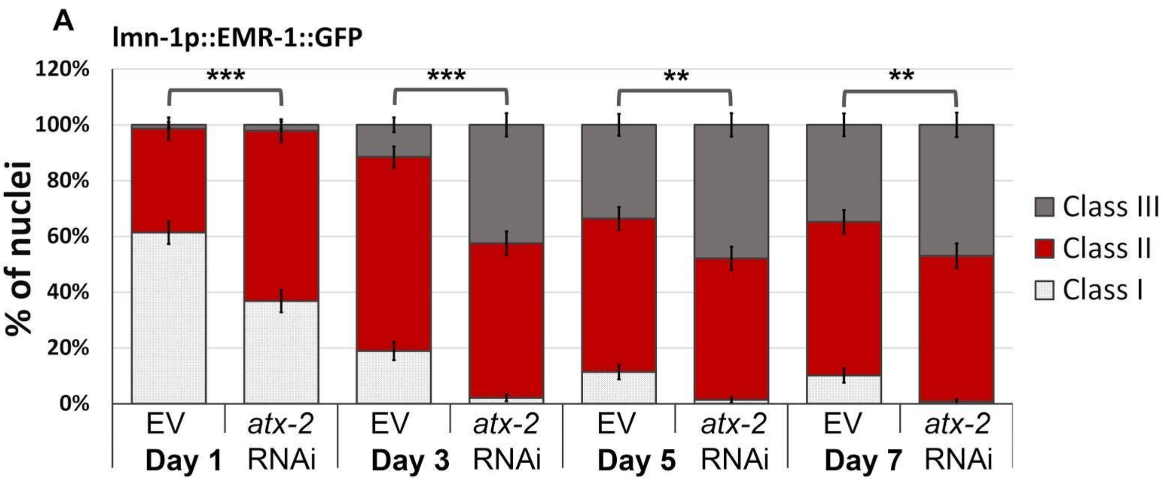

Fig. S3. ATX-2 is required to maintain nuclear structure. Relative distribution of the 3 different classes grading the nuclear morphology changes in EMR-1::GFP expressing worms fed with *atx-2* or EV at days 1, 3, 5 and 7 of adulthood. n(D1)=289 nuclei, n(D3)=287 nuclei, n(D5)=291 nuclei, n(D7)=268 nuclei. Error bars in all graphs represent mean ± SEM. P value was calculated using Fisher Exact Probability Test.

Table S1. Primer pairs used for real-time PCR.

Name	Sequence
lmn-1 F1	AACTTGGCCGATCGCTTC
lmn-1 R1	GAGCTCATCTTGAGCCGAAT
lmn-1 F2	TCGCTAAGCAACAATGGAGG
lmn-1 R2	GCGAATTTGAACCTGGAGTC
atx-2 F1	GCAGCAGCAACACATTCAAC
atx-2 R1	TCTGTTGCATTGGCATCTGT
atx-2 F2	AGTATATGGTGATGCAGGGAC
atx-2 R2	CGCTCGCCCATAAGACTTTG
daf-15 F1	GGCGATTCATGCAATGGAAG
daf-15 R1	GGGCAGAATGGATTTGAAGC
daf-15 F2	GCTCGATCGCTAACCACTTC
daf-15 R2	TTGGCGGATAATTGTCATCA
rheb-1 F1	AGATCACGGCCAGAGAATCG
rheb-1 R1	TCTCACGGAGAAGCAATTCG
rheb-1 F2	AATGGGATGCGAAGTTTGTC
rheb-1 R2	TTCGAACACCTCATGCACTC
aak-2 F1	CAAGAGTTTGGCCGACGAGG
aak-2 R1	TGATGCGTGTCGGGATGAAG
aak-2 F2	TTGTTGGATTTCAAGAGTTTGG
aak-2 R2	TTCTGCGGCATAGACATTGA
pmp-3 F	gttcccgtgttcatcactcat
pmp-3 R	acaccgtcgagaagctgtaga
act-1 F1	gctggacgtgatcttactgattacc
act-1 R1	gtagcagagcttctccttgatgtc

Journal of Cell Science • Supplementary information
