# Supplementary material for: Lamin regulates the dietary restriction response via the mTOR pathway in Caenorhabditis elegans
Source: J Cell Sci. 2021 Sep 6;134(17):jcs258428. doi: 10.1242/jcs.258428 (PMC8445603; doi:10.1242/jcs.258428)
Supplement: Reviewer comments [file joces-134-258428_review_history.pdf]

# Lamin regulates the dietary restriction response via the mTOR pathway in *Caenorhabditis elegans*

Chayki Charar, Sally Metsuyanim-Cohen and Daniel Z. Bar
DOI: 10.1242/jcs.258428

**Editor**: Maria Carmo-Fonseca

**Review timeline**
Original submission: 4 February 2021
Editorial decision: 15 March 2021
First revision received: 26 June 2021
Editorial decision: 20 July 2021
Second revision received: 29 July 2021
Accepted: 30 July 2021

---

**Original submission**

First decision letter

MS ID#: JOCES/2021/258428

MS TITLE: Lamin regulates the dietary restriction response via the mTOR pathway in Caenorhabditis elegans

AUTHORS: Daniel Z Bar, Chayki Charar, and Sally Metsuyanim-Cohen
ARTICLE TYPE: Research Article

We have now reached a decision on the above manuscript.

To see the reviewers' reports and a copy of this decision letter, please go to: https://submit-jcs.biologists.org and click on the 'Manuscripts with Decisions' queue in the Author Area. (Corresponding author only has access to reviews.)

As you will see, both reviewers thought that the work was potentially quite interesting and significant but all also raised a number of concerns that must be dealt with before the manuscript can be reconsidered.
Please address these issues as thoroughly as possible. In particular, you should show what the knockdown efficiency is by real-time RT PCR. Otherwise, I consider that no further experiments are required. Rather, you should revise the manuscript taking into account the reviewers' comments.
If you think that you can deal satisfactorily with the criticisms on revision, I would be pleased to see a revised manuscript.

*We are aware that you may be experiencing disruption to the normal running of your lab that makes experimental revisions challenging. If it would be helpful, we encourage you to contact us to discuss your revision in greater detail. Please send us a point-by-point response indicating where you are able to address concerns raised (either experimentally or by changes to the text) and where you will not be able to do so within the normal timeframe of a revision. We will then provide further guidance. Please also note that we are happy to extend revision timeframes as necessary.*

© 2021. Published by The Company of Biologists under the terms of the Creative Commons Attribution License (https://creativecommons.org/licenses/by/4.0/).

Please ensure that you clearly highlight all changes made in the revised manuscript. Please avoid using 'Tracked changes' in Word files as these are lost in PDF conversion.

I should be grateful if you would also provide a point-by-point response detailing how you have dealt with the points raised by the reviewers in the 'Response to Reviewers' box. Please attend to all of the reviewers' comments. If you do not agree with any of their criticisms or suggestions please explain clearly why this is so.

Reviewer 1

*Advance summary and potential significance to field*

Animals under DR conditions have reduced size. Bar et al., have previously identify a novel mechanism that links the mTOR pathway as well as previously unknown regulators of the mTOR pathway to cell size regulation and DR. In this manuscript "Lamin regulates the dietary restriction response via the mTOR pathway in Caenorhabditis elegans" by Charar et al they explore this link further and demonstrate that lamin is a regulator of DR, specifically cell size, acting via the mTOR pathway. This link is intriguing given the known role of lamin in progeria and its link to mTOR dysregulation. Charar et al utilise C. elegans to explore the underlying mechanism linking DR, lamin and mTOR.

*Comments for the author*

Required revisions:
(1) For the epistasis analyses (Figures 3-4): The authors need to show what the knockdown efficiency is by real-time RT PCR. This is specifically important in the double RNAi exp (Figs. 3A. 3C, 4A and 4B) as the efficiency of double RNAi was shown to be lower for each gene which can impact the interpretation of the epistasis relationships. In general mutants rather than knockdowns are required for epistasis conclusions, see Gems D et al Aging Cell 2002).
(2) eat-2 is a well established genetic model of DR. However, there are differences between DR protocols and downstream pathway and it is good (and accepted) practice to use additional DR treatments to make general statements about DR regulation. It will aide the manuscript if at least the impact of lmn-1 RNAi on DR-dependent cell size was demonstrated by another DR regime. Alternately, please note in the discussion that the conclusions are based genetic model of DR.
(3) There is no information on how the western was performed in the method section. Was the exp in Fig. 5H performed in triplicates as suggested in the statistic section? If so why there are no error bars in Fig. 5I? To claim that LMN-1 levels are modulated with age at least triplicates are required.

Minor comments:
(1) the Friedland et al. 2013 in the method section does not appear in the reference list. Please check refs.
(2) Immunofluorescence: it is stated that "Samples were incubated for 25 min in X2 Modified Ruvkun's Witches Brew (MRWB) ("Gonad-intestine Staining Protocol," n.d.)" Please give details of ref.
(3) Please add a section on western in the method section.

Reviewer 2

*Advance summary and potential significance to field*

This paper is unique and important. The authors used rigorous C. elegans genetics to answer a most challenging question: whether and how lamins (nuclear intermediate filaments) influence the mTORC1 pathway (global metabolic control). The answer to this question is a definitive 'yes'; genetic epistasis places lamin upstream of both Raptor (mTOR complex component) and S6kinase (downstream target and effector). In the context of dietary restriction, lamin is required to block the nuclear entry of RAGC-1(GTP), which must enter the nucleus to achieve the GDP-bound state before returning to the cytoplasm to activate mTORC1. These results open new doors to exploring

© 2021. Published by The Company of Biologists under the terms of the Creative Commons Attribution License (https://creativecommons.org/licenses/by/4.0/). 

and understanding fundamental biology and the mechanisms of human diseases caused by mutations in lamins.

*Comments for the author*

The manuscript is terse (tightly genetic) and very clearly written. The results are focused, well-controlled and convincing. Two or three text/graph revisions are required.
Revision-1 (required): The discussion is inexplicably biased towards a model that focuses on nuclear pore complexes (NPC), e.g., loss of lamins alters mechanical control of NPC structure/function. The discussion must consider an equally plausible alternative model, e.g., lamin depletion also causes mislocalization and functional loss of emerin and other inner nuclear membrane (INM) proteins; emerin and related proteins are known to dampen signaling transcription factors (e.g., ERK1/2 and b-catenin, etc) by somehow 'ejecting' them from the nucleus. If the authors have evidence that favors their NPC model, they should show it. Otherwise, discuss all plausible models.
Revision-3 (required): The colors used in Figures 1B/D, 2B/D, 3ABC, 4CDEF, 5G, 6I, 7D, and supplemental figure 2G and 3 are unacceptable because they're indistinguishable when printed in black/white/grayscale. Revise to black/white/grayscale-compatible colors.
Revision-4 (requested; would significantly enhance manuscript): Add a model figure to summarize these findings and indicate where new questions emanate.
Minor corrections (counting Title page as Page-1):
Page 3, first paragraph, near bottom:
(a) change "Downregulated for lmn-1 increase" to "Downregulation of lmn-1 increased". (b) change "constitute the increase" to "are responsible for the increase"
Page 4: Add a reference for this sentence: "ATX-2 regulates the mTOR..."
Acknowledgements: Indicate which scientist was principal investigator of the ISF grant.

---

**First revision**

<u>Author response to reviewers' comments</u>

Reviewer 1 Advance Summary and Potential Significance to Field:
Animals under DR conditions have reduced size. Bar et al., have previously identify a novel mechanism that links the mTOR pathway as well as previously unknown regulators of the mTOR pathway to cell size regulation and DR. In this manuscript "Lamin regulates the dietary restriction response via the mTOR pathway in Caenorhabditis elegans" by Charar et al they explore this link further and demonstrate that lamin is a regulator of DR, specifically cell size, acting via the mTOR pathway. This link is intriguing given the known role of lamin in progeria and its link to mTOR dysregulation. Charar et al utilise C. elegans to explore the underlying mechanism linking DR, lamin and mTOR.

We thank the reviewer for the comments and insights.

Reviewer 1 Comments for the Author:
Required revisions:
(1) For the epistasis analyses (Figures 3-4): The authors need to show what the knockdown efficiency is by real-time RT PCR. This is specifically important in the double RNAi exp (Figs. 3A. 3C, 4A and 4B) as the efficiency of double RNAi was shown to be lower for each gene which can impact the interpretation of the epistasis relationships. In general mutants rather than knockdowns are required for epistasis conclusions, see Gems D et al Aging Cell 2002).

We completely agree that mutants and full deletions are preferable, and use them when possible. However, many of these genes are essential, and thus don't have stable null/loss-of-function lines, or have balanced heterozygous lines that in our hands provide too much variability across multiple phenotypes in the (sterile) null progeny.

As suggested, we thus performed the real time experiments that are now available as

© 2021. Published by The Company of Biologists under the terms of the Creative Commons Attribution License (https://creativecommons.org/licenses/by/4.0/). 

supplementary figure 1B.

(2) eat-2 is a well established genetic model of DR. However, there are differences between DR protocols and downstream pathway and it is good (and accepted) practice to use additional DR treatments to make general statements about DR regulation. It will aide the manuscript if at least the impact of lmn-1 RNAi on DR-dependent cell size was demonstrated by another DR regime. Alternately, please note in the discussion that the conclusions are based genetic model of DR.

Thank you for this important comment. While we previously validated that some genes in this pathway work on additional DR mutants, as well as using bacterial dilution, we have not done this to all the genes explored in this manuscript. Thus, we adjusted the discussion section to state this fact and limit our findings.

(3) There is no information on how the western was performed in the method section. Was the exp in Fig. 5H performed in triplicates as suggested in the statistic section? If so why there are no error bars in Fig. 5I? To claim that LMN-1 levels are modulated with age at least triplicates are required.

We updated the methods section to include the WB details. These WBs were performed 3 times, two experiments were done in parallel and one independently at a different time point. This author is very cautious about giving numerical values to WBs. Our claim: “... a decrease in total LMN-1 levels from day 2 to day 4 in control animals, and a smaller decrease from day 4 to day 6 in DR animals“ aimed to reflect the previously reported finding that levels of lamin mildly decrease with age (Please see Haithcock et al., 2005, Supp fig 8, that shows a decrease in LMN-1 and to a much lesser extent of the LMN-1/actin ratio) and to show that no drastic changes to LMN-1 abundance are observed in eat-2 animals. These observations are also supported by the microscopy data. We thus adjusted the text and removed the quantification part (5I).

Minor comments:
(1) the Friedland et al. 2013 in the method section does not appear in the reference list. Please check refs.
Corrected.
(2) Immunofluorescence: it is stated that "Samples were incubated for 25 min in X2 Modified Ruvkun's Witches Brew (MRWB) (“Gonad-intestine Staining Protocol,” n.d.)" Please give details of ref.
Corrected.

(3) Please add a section on western in the method section.
Added.

Reviewer 2 Advance Summary and Potential Significance to Field:
This paper is unique and important. The authors used rigorous C. elegans genetics to answer a most challenging question: whether and how lamins (nuclear intermediate filaments) influence the mTORC1 pathway (global metabolic control). The answer to this question is a definitive 'yes'; genetic epistasis places lamin upstream of both Raptor (mTOR complex component) and S6kinase (downstream target and ffector). In the context of dietary restriction, lamin is required to block the nuclear entry of RAGC-1(GTP), which must enter the nucleus to achieve the GDP-bound state before returning to the cytoplasm to activate mTORC1. These results open new doors to exploring and understanding fundamental biology and the mechanisms of human diseases caused by mutations in lamins.

We thank the reviewer for these kind words.

Reviewer 2 Comments for the Author:
The manuscript is terse (tightly genetic) and very clearly written. The results are focused, well-controlled and convincing. Two or three text/graph revisions are required.

Revision-1 (required): The discussion is inexplicably biased towards a model that focuses on nuclear pore complexes (NPC), e.g., loss of lamins alters mechanical control of NPC structure/function. The discussion must consider an equally plausible alternative model, e.g., lamin depletion also causes

© 2021. Published by The Company of Biologists under the terms of the Creative Commons Attribution License (https://creativecommons.org/licenses/by/4.0/).

mislocalization and functional loss of emerin and other inner nuclear membrane (INM) proteins; emerin and related proteins are known to dampen signaling transcription factors (e.g., ERK1/2 and b-catenin, etc) by somehow 'ejecting' them from the nucleus. If the authors have evidence that favors their NPC model, they should show it. Otherwise, discuss all plausible models.
We agree with this criticism, and significantly expanded the discussion section to include other possibilities. Specifically for Emerin, we previously tested the downregulation of *emr-1* with and without *lem-2,* and found no effect on animal size (in *eat-2* nematodes).

Revision-3 (required): The colors used in Figures 1B/D, 2B/D, 3ABC, 4CDEF, 5G, 6I, 7D, and supplemental figure 2G and 3 are unacceptable because they're indistinguishable when printed in black/white/grayscale. Revise to black/white/grayscale-compatible colors.
All bars are now clearly distinguishable when printed in grayscale.

Revision-4 (requested; would significantly enhance manuscript): Add a model figure to summarize these findings and indicate where new questions emanate.
A model figure is now available as Fig. 8.

Minor corrections (counting Title page as Page-1):
Page 3, first paragraph, near bottom:
(a) change "Downregulated for lmn-1 increase" to "Downregulation of lmn-1 increased". (b) change "constitute the increase" to "are responsible for the increase"
Corrected.

Page 4: Add a reference for this sentence: "ATX-2 regulates the mTOR..."
Added.

Acknowledgements: Indicate which scientist was principal investigator of the ISF grant.
Added.

---

Second decision letter

MS ID#: JOCES/2021/258428

MS TITLE: Lamin regulates the dietary restriction response via the mTOR pathway in Caenorhabditis elegans

AUTHORS: Daniel Z Bar, Chayki Charar, and Sally Metsuyanim-Cohen
ARTICLE TYPE: Research Article

We have now reached a decision on the above manuscript.

To see the reviewers' reports and a copy of this decision letter, please go to: https://submit-jcs.biologists.org and click on the 'Manuscripts with Decisions' queue in the Author Area. (Corresponding author only has access to reviews.)

As you will see, the reviewers recognize that most of their initial criticisms have been addressed in your revised manuscript. However, reviewer #2 still raised issues that will require amendments to your manuscript. I hope that you will be able to carry these out, because I would like to be able to accept your paper.

*We are aware that you may be experiencing disruption to the normal running of your lab that makes experimental revisions challenging. If it would be helpful, we encourage you to contact us to discuss your revision in greater detail. Please send us a point-by-point response indicating where you are able to address concerns raised (either experimentally or by changes to the text) and where you will not be able to do so within the normal timeframe of a revision. We will then*

© 2021. Published by The Company of Biologists under the terms of the Creative Commons Attribution License (https://creativecommons.org/licenses/by/4.0/). 

*provide further guidance. Please also note that we are happy to extend revision timeframes as necessary.*

Please ensure that you clearly highlight all changes made in the revised manuscript. Please avoid using 'Tracked changes' in Word files as these are lost in PDF conversion.

I should be grateful if you would also provide a point-by-point response detailing how you have dealt with the points raised by the reviewers in the 'Response to Reviewers' box. Please attend to all of the reviewers' comments. If you do not agree with any of their criticisms or suggestions please explain clearly why this is so.

Reviewer 1

*Advance summary and potential significance to field*

"Lamin regulates the dietary restriction response via the mTOR pathway in Caenorhabditis elegans" by Charar et al explores the link between mTOR pathway cell size regulation and DR and demonstrates that lamin is a regulator of DR specifically cell size, acting via the mTOR pathway.

*Comments for the author*

The authors responded to all my comments, and I now recommend the manuscript for publication in JCS.

Reviewer 2

*Advance summary and potential significance to field*

This important paper significantly advances our understanding of the interplay between nuclear lamin filaments and aging/longevity, by showing that lamins control mTOR activation (directly or indirectly) by regulating the nuclear entry, hence the GTP-vs-GDP status, of RGC-1.

*Comments for the author*

These revisions satisfy previous concerns; however, the manuscript requires redrawing of new Figure 8 (model), and minor text revisions.
Figure 8 is messy and unclear.
(a) Misspelled “Wilde Type”-- change to “Wild Type” or “Wildtype”.
(b) Draw the nuclear envelope correctly (two membranes that connect at NPCs).
(c) Change both greens, and red, to colors that will be visible to red/green colorblind people.
(d) Add the words “active” and “inactive” below TORC1.
(e) Make the model elegant by showing (a) fewer copies of RGC-1/GTP and RGC-1/GDP, and (b) fewer and better-looking in/out arrows in Wildtype.
Text corrections/revisions:
Page 3, first paragraph: change “Downregulated for lmn-1” to “Downregulation of lmn-1”
Page 7: Add ‘protein’ to this line: total LMN-1 protein levels in control and DR animals with age (Fig. 5H)...”
New discussion, page 11: change “multiple nuclear lamina proteins” to “multiple nuclear membrane proteins”.

---

**Second revision**

Author response to reviewers' comments

© 2021. Published by The Company of Biologists under the terms of the Creative Commons Attribution License (https://creativecommons.org/licenses/by/4.0/).

We thank the reviewers for helping make this a better manuscript. All the suggestions/corrections were accepted and incorporated into the manuscript.

---

Third decision letter

MS ID#: JOCES/2021/258428

MS TITLE: Lamin regulates the dietary restriction response via the mTOR pathway in Caenorhabditis elegans

AUTHORS: Daniel Z Bar, Chayki Charar, and Sally Metsuyanim-Cohen
ARTICLE TYPE: Research Article

I am happy to tell you that your manuscript has been accepted for publication in Journal of Cell Science, pending standard ethics checks.

© 2021. Published by The Company of Biologists under the terms of the Creative Commons Attribution License (https://creativecommons.org/licenses/by/4.0/).
